# DCPA-SNN, Direct-Coding-Physics-Aware Spiking Neural Network: A Framework for Wearable ECG Denoising Under Dynamic-Noise Conditions

**DOI:** 10.3390/s26154695

**Published:** 2026-07-23

**Authors:** Yukun Ren, Hongyou Zuo, Yuhang Cai, Shenghua Wang, Guihao Ran, Dakun Lai

**Affiliations:** 1 Glasgow College, University of Electronic Science and Technology of China, Chengdu 611731, China; 2 School of Electronic Science and Engineering, University of Electronic Science and Technology of China, Chengdu 611731, China

**Keywords:** wearable ECG, denoising, spiking neural network, edge computing, energy consumption

## Abstract

Smart wearable electrocardiogram (ECG) monitoring enables continuous cardiac assessment beyond clinical settings, but ECG signals are often degraded by baseline wander, muscle artifacts, and electrode motion artifacts. At present, pure end-to-end spiking neural networks (SNNs), with the advantage of low computational complexity, have rarely been explored for ECG noise suppression, particularly under dynamic conditions. To address this gap, this study proposes Direct-Coding-Physics-Aware Spiking Neural Network (DCPA-SNN) for wearable ECG denoising. The proposed method integrates a direct-coding SNN, channel attention, residual noise learning, and a physics-aware multi-domain loss function to preserve diagnostically important waveform structures. Clean ECG signals from the MIT-BIH Arrhythmia Database and real-noise segments from the MIT-BIH Noise Stress Test Database were used to construct single-noise and mixed-noise evaluation scenarios with input SNRs ranging from −6 dB to 4 dB to reflect the noise characteristics of wearable devices. Experimental results demonstrate that DCPA-SNN achieves robust denoising performance under different noise conditions. In the mixed-noise scenario, which serves as the primary evaluation setting of this study, the average denoised SNR reached 5.80 dB, with an average SNR improvement of 6.80 dB, while the R-peak detection rate increased from 90.71% to 95.72%. These results demonstrate that the proposed model, DCPA-SNN, provides a promising approach for wearable ECG denoising with potential for low-power deployment.

## 1. Introduction

Cardiovascular diseases (CVDs) have become one of the leading causes of death and a major threat to human life and health worldwide. According to data released by the World Health Organization, approximately 19.8 million people died from CVDs globally in 2022, accounting for 32% of all deaths [1]. The Report on Cardiovascular Health and Diseases in China 2024 further indicates that the incidence and mortality of cardiovascular diseases in China will continue to rise over the next decade [2]. Therefore, the development of early-stage, non-invasive, and long-term electrocardiogram (ECG) monitoring technologies is of great significance for the prevention and clinical diagnosis of cardiovascular diseases.

Wearable electrocardiogram (ECG) monitoring systems have evolved from conventional portable sensing devices to smart wearable ECG systems integrated with real-time signal processing and intelligent analytics, enabling long-term, non-invasive cardiac health assessment in remote- and continuous-monitoring scenarios [3]. Compared with conventional short-term clinical ECG acquisition, smart wearable ECG devices are typically deployed in daily-life environments, where subject motion, respiration, perspiration, and electrode–skin impedance variations introduce significant disturbances [4,5]. These factors generate strong non-stationary noise components, leading to severe degradation of signal quality and potentially compromising downstream tasks such as rhythm analysis, arrhythmia detection, and clinical interpretation [6]. The major types of noise, including baseline wander (BW), muscle artifact (MA), and electrode motion artifact (EM), are summarized in Table 1 [7,8]. In recent years, wearable ECG systems have evolved toward smart and intelligent monitoring that integrates embedded machine learning for real-time signal quality assessment and cardiac event detection. This development has accelerated the adoption of edge computing for on-device ECG processing, reducing reliance on cloud transmission [9]. However, a key trade-off remains between the increasing computational complexity of intelligent ECG algorithms and the limited power, memory, and processing capability of wearable devices, which constrains real-time and energy-efficient deployment in practical scenarios [10].

To address these issues, extensive studies have been conducted on ECG denoising, mainly following two technical routes: traditional signal-processing methods and deep learning-based methods. Traditional approaches, such as wavelet-threshold denoising, empirical mode decomposition (EMD), and adaptive filtering, have been widely applied due to their low computational complexity and ease of implementation [11]. However, under low signal-to-noise ratio (SNR) conditions, these methods often suffer from insufficient denoising or from distortion of effective features. For example, wavelet-based methods can lead to the loss of critical morphological information when the threshold is improperly selected [12]; EMD is prone to mode mixing when processing signals with close frequency components, which may weaken or even filter out important features such as P waves and T waves [13]. Meanwhile, deep learning methods have also demonstrated superior performance in ECG denoising. Convolutional neural networks (CNNs) enable end-to-end modeling but remain limited in capturing long-range temporal dependencies [14]. Transformer-based models have advantages in modeling global dependencies; however, their large parameter scale and high computational complexity make them difficult to deploy on resource-constrained wearable devices [15]. In addition, some studies have introduced graph neural networks (GNNs) to enhance signal representation capability, but the high computational overhead of graph convolution operations likewise limits their real-time applicability [16].

ECG denoising methods specifically designed for wearable signals remain scarce. Material and structural optimizations, such as motion-resistant materials [17], textile-based electrodes [18,19,20], electronic-skin electrodes [21], and microneedle-array electrodes [22], improve contact but cannot remove non-stationary motion noise. Direct application of conventional denoising methods [23] or auxiliary-sensor approaches [24] often yields limited performance or increases system complexity.

Such noise frequently overlaps with key ECG features (P waves, QRS complexes, and ST segments) and exhibits transient, burst-like, event-driven characteristics, making it challenging to model while respecting wearable devices’ constraints on computation, memory, and power [6].

To address these challenges, this paper proposes Direct-Coding-Physics-Aware Spiking Neural Network (DCPA-SNN), a model for wearable ECG denoising under dynamic-noise conditions, treating it as an event-driven signal reconstruction problem. The model incorporates feature-adaptive enhancement and a physics-aware multi-domain loss to preserve diagnostically relevant waveform structures while retaining the potential for low-power implementation. Compared with conventional continuous-valued networks, the proposed framework may be more compatible with dynamic wearable ECG monitoring scenarios due to its event-driven computation mechanism. The main contributions are summarized as follows:

1. SNN-based ECG denoising framework: To the best of our knowledge, this work explores an end-to-end SNN-based framework specifically designed for wearable ECG denoising, providing a preliminary attempt to address dynamic-noise suppression using event-driven neural computation.
2. Direct-coding spiking neural network: A direct-coding strategy for the spiking neural network is designed, enabling the model to form differentiated internal representations of effective ECG features and noise perturbations.
3. Clinical-diagnosis-oriented consistency optimization: A physics-aware multi-domain joint loss function is proposed to enhance the preservation of key diagnostic waveforms.
4. High-energy-efficiency design for wearable applications: The sparse event-driven computational characteristics of spiking neural networks are exploited to improve the deployment potential of the proposed model in resource-constrained wearable devices and neuromorphic computing platforms.

## 2. Materials and Methods

### 2.1. Framework

**Figure 1:**
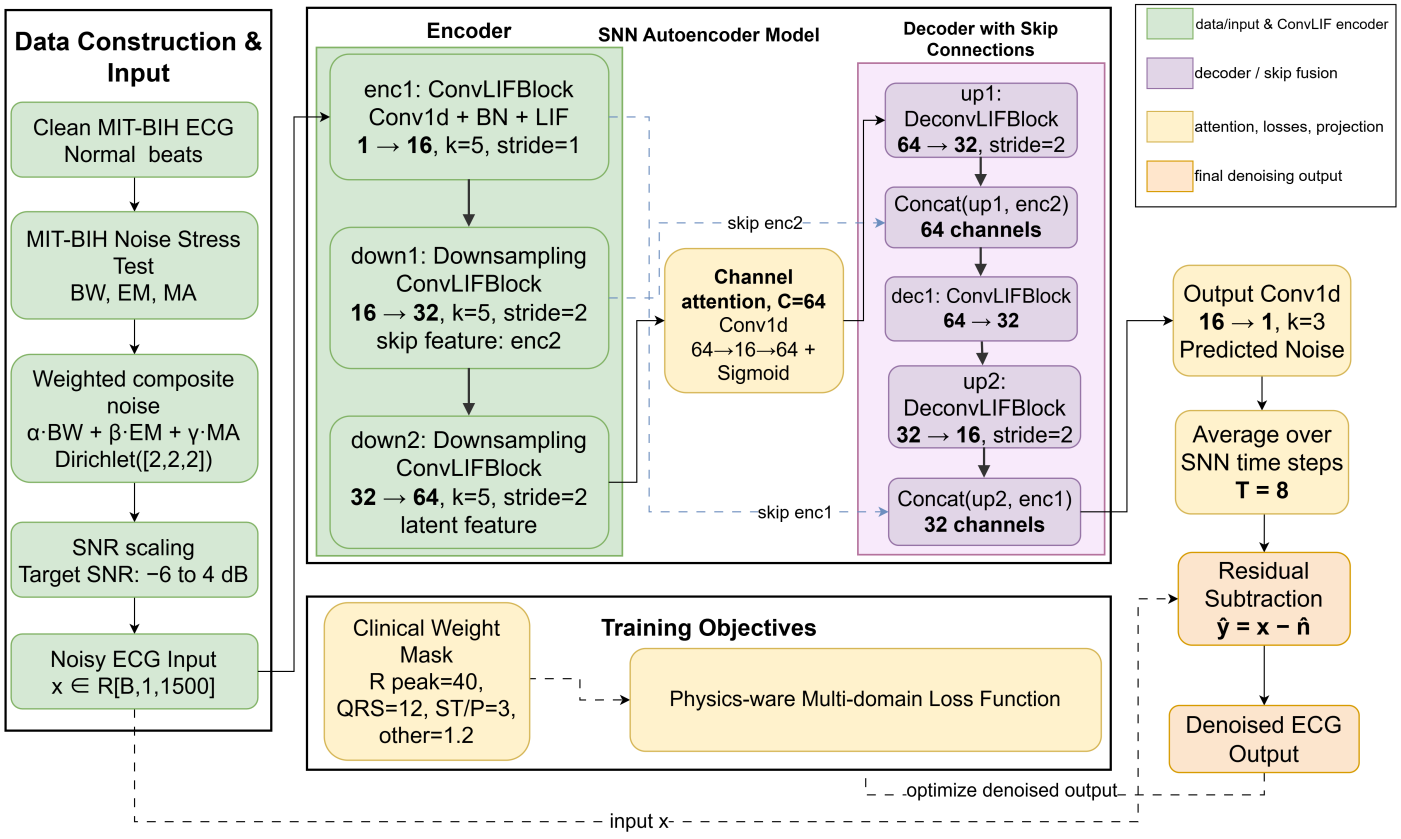
Framework of the proposed model.

Figure 1 illustrates the proposed denoising framework, DCPA-SNN. Built upon spiking neural networks (SNNs), this framework integrates physics-aware modeling and multi-domain joint strategies and adopts a channel attention mechanism to denoise noisy electrocardiogram (ECG) signals acquired under dynamic monitoring conditions. The overall workflow consists of three core stages: signal preprocessing, the training of the SNN-based feature extraction model, and the denoising of noisy ECG signals. The black arrows in the figure indicate the operating flow of the system.

DCPA-SNN processes noisy wearable ECG signals using an encoder–decoder SNN equipped with channel attention (Figure 1). The encoder extracts and enhances informative features, while the U-Net-inspired decoder estimates the noise component; the clean ECG signal is subsequently recovered through residual subtraction. This architecture further exploits the temporal integration, threshold-triggered firing, and state evolution of LIF neurons to capture dynamic, non-stationary noise and transient waveform variations [25].

### 2.2. Dataset

The training, validation, and test sets were constructed from two public benchmark databases. Clean ECG signals were obtained from the MIT-BIH Arrhythmia Database [26], while real clinical noise was derived from the MIT-BIH Noise Stress Test Database [27]. The MIT-BIH Arrhythmia Database contains 48 two-channel ambulatory ECG recordings with an original sampling frequency of 360 Hz and expert-annotated R peaks [26]. All ECG signals were resampled to 250 Hz to match the sampling requirements of wearable ECG devices, and the R-peak annotations were scaled accordingly to maintain temporal consistency. In this study, four paced records were excluded, and the remaining 44 non-paced records were used for model development and evaluation. To avoid record-level data leakage, a strict cross-record partitioning strategy was adopted. Specifically, 28 records were used for model training; 8 records were used as the validation set for learning-rate scheduling, early stopping, and best-checkpoint selection; and the remaining 8 records were held out as an independent test set for final performance evaluation. The test records were not used during model training, hyperparameter adjustment, learning-rate scheduling, or checkpoint selection.

### 2.3. Direct-Coding-Physics-Aware Spiking Neural Networks

#### 2.3.1. Rationale for Using SNNs

Spiking neural networks (SNNs) are regarded as the third generation of artificial neural networks. Owing to their biologically inspired computing paradigm, SNNs possess inherent spatiotemporal information processing capability and extremely low hardware energy consumption [28,29].

SNNs are naturally well suited to ECG denoising tasks for several reasons. First, ECG signals are intrinsically temporal physiological signals, and the membrane-potential integration mechanism of SNNs can naturally model long-range temporal dependencies, enabling efficient temporal feature extraction without introducing additional recurrent structures [28,30]. Second, SNNs can effectively capture long-range temporal dependencies in non-stationary signals, thereby providing stronger modeling capability for non-stationary noise components such as baseline wander and muscle artifacts than conventional convolutional neural networks (CNNs) [29,30]. Third, the sparse computation property of SNNs enables a reduction in energy consumption by one to two orders of magnitude on neuromorphic hardware compared with conventional deep learning models, making them highly compatible with the low-power requirements of wearable ECG devices [29]. In this study, we fully exploit the advantages of SNNs in event-driven modeling and sparse computation and further optimize their network architecture and key modules for the ECG denoising task.

#### 2.3.2. Channel Attention Mechanism

Research indicates that different feature channels within ECG signals contribute differently to the final denoising performance. Certain channels may primarily respond to key waveform structures—such as the QRS complex, ST segment, and P wave—whereas others may predominantly capture noise perturbations. If the model uniformly assigns equal importance to all channels, it may lead to an insufficient representation of crucial waveform features or the erroneous amplification of noise-related characteristics.

To address these issues, this study introduces a channel attention mechanism at the bottleneck layer of the spiking neural network (SNN) autoencoder. This mechanism dynamically learns the weights of different channels based on the response of the input ECG features, thereby selectively enhancing the valid features associated with critical waveforms. Introducing the attention mechanism at the bottleneck layer facilitates dimensionality adjustment and reduces computational complexity [31,32]. After multiple layers of convolution and downsampling, the bottleneck features have inherently fused a broad range of temporal contextual information, enabling a more comprehensive representation of the global waveform structure of the ECG signal [33]. Consequently, executing channel recalibration at this specific layer allows for a more effective distinction between valid electrocardiographic features and noise-dominated components. Furthermore, because the temporal dimension at this stage is significantly reduced, deploying the attention mechanism here incurs substantially lower computational overhead compared with applying it to shallower feature maps [34].

#### 2.3.3. Physics-Aware Multi-Domain Loss Function

For ECG denoising tasks, preserving diagnostically relevant waveform structures is important for downstream ECG interpretation. In electrocardiogram signals, the QRS complex, ST segment, and P wave contain essential information for the diagnosis of arrhythmias, myocardial ischemia, conduction abnormalities, and other cardiovascular diseases [35,36]. However, conventional mean squared error (MSE)-based optimization assigns identical weights to all sampling points, thereby neglecting the varying clinical significance of different ECG waveform regions [13]. Consequently, a model optimized solely with reconstruction error may achieve favorable numerical performance while failing to preserve diagnostically important waveform characteristics.

To address this issue, a physics-aware multi-domain loss function is proposed. The overall training objective constrains the network from multiple perspectives and is formulated as:

(1) $$\begin{array}{cc} L_{\mathrm{cmd}}= & \;L_{\mathrm{cw}}+\lambda_{1}L_{1}+\lambda_{p}L_{p}+\lambda_{f}L_{f}+\lambda_{qrs}L_{qrs}+\lambda_{r}L_{r}\\ \; & +\alpha \left(e\right)\left(\lambda_{d}L_{d}+\lambda_{tv}L_{tv}\right), \end{array}$$

where $L_{\mathrm{cw}}$ denotes the clinically weighted reconstruction loss. The other terms correspond to $L_{1}$ reconstruction ($L_{1}$), perceptual ($L_{p}$), frequency-domain consistency ($L_{f}$), QRS slope preservation ($L_{qrs}$), R-peak amplitude preservation ($L_{r}$), differential smoothness ($L_{d}$), and total variation ($L_{tv}$) losses. The empirical weighting coefficients are set to $\lambda_{1}=0.5$, $\lambda_{p}=0.1$, $\lambda_{f}=0.05$, $\lambda_{qrs}=0.3$, $\lambda_{r}=2.0$, $\lambda_{d}=0.12$, and $\lambda_{tv}=0.02$. The term α(e) is an epoch-dependent scheduling factor that prevents the network from generating excessively smoothed waveforms before meaningful physiological features are learned.

Among all loss components, $L_{\mathrm{cw}}$ serves as the core optimization objective to maximize the preservation of diagnostically significant structures. It is defined as:

(2) $$L_{\mathrm{cw}}=\frac{1}{N}\sum_{i=1}^{N}w_{i}{\left({\hat{y}}_{i}-y_{i}\right)}^{2},$$

where *N* denotes the sequence length, ${\hat{y}}_{i}$ and $y_{i}$ represent the denoised and clean signals at the *i*-th sampling point, and $w_{i}$ is the position-specific clinical weight. Based on annotated R-peak locations, $w_{i}$ is assigned according to regional clinical relevance:

(3) $$w_{i}=\left\{\begin{array}{cc} 12.0, & i\in \Omega_{\mathrm{QRS}}\\ 3.0, & i\in \Omega_{\mathrm{ST}}\cup \Omega_{P}\\ 1.5, & i\in \Omega_{\mathrm{smooth}}\\ 1.2, & \mathrm{otherwise} \end{array}\right.$$

where $\Omega_{\mathrm{QRS}}$, $\Omega_{\mathrm{ST}}$, $\Omega_{P}$, and $\Omega_{\mathrm{smooth}}$ denote the QRS complex, ST segment, P wave, and low-gradient smooth regions, respectively. It should be noted that the clinical weight mask is used only during supervised training to construct the physics-aware loss. During inference, the trained DCPA-SNN model requires only the noisy ECG segment as input and does not require clean ECG signals, R-peak annotations, or clinical weight masks.

Hyperparameter Selection and Sensitivity Analysis: The weighting coefficients ($\lambda_{1},\;\lambda_{qrs},\;\lambda_{r}$, etc.) in the physics-aware loss function were selected empirically via grid-based validation tuning to balance global noise reduction and local clinical morphology preservation. In particular, higher weights were assigned to R-peak and QRS-related regions to preserve R-wave amplitude and QRS slope, while moderate weights were used for ST/P-related regions to reduce the over-smoothing of low-amplitude diagnostic components. Following systematic design optimization principles (as discussed in [37]), candidate coefficient combinations were evaluated using validation loss, SNR improvement, QRS-region SNR, ST/P-region SNR, and waveform visualization. Thus, the selected coefficients were determined based on validation performance and preliminary experiments. To systematically reduce the multiparameter search burden in future extensions, Design-of-Experiments (DoE) methods such as Taguchi optimization will be incorporated.

## 3. Experiments

### 3.1. Experimental Design and Noise Superposition

**Figure 2:**
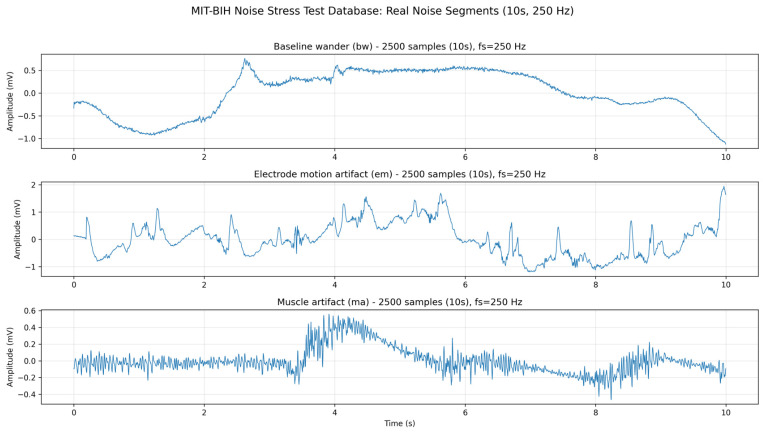
Three examples of noise: BW, EM and MA.

The noisy ECG dataset was constructed by superimposing real clinical noise onto clean ECG signals [27], rather than using synthetic Gaussian noise, to better simulate wearable monitoring scenarios. Three representative noise types—baseline wander (BW), muscle artifact (MA), and electrode motion (EM)—were extracted from the MIT-BIH Noise Stress Test Database and are illustrated in Figure 2. Noise intensity was controlled using an energy-ratio-based SNR scaling method, and R-peak annotations were employed to generate clinical feature-weighted masks, assigning higher weights to diagnostically important regions including the QRS complex, ST segment, and P wave.

Three single-noise test sets were constructed for BW, EM, and MA to evaluate the noise-specific denoising capability of the proposed model. All test samples were generated from the independent test set records isolated from training, with sample length and sampling rate consistent with the main experiment. The SNR range was set from −6 dB to 4 dB to reflect realistic wearable scenarios [38], and each set contained 2000 independent samples generated using the same energy-ratio-based method.

To assess robustness under complex noise conditions, a mixed-noise test set was constructed following [14,39,40]. BW, MA, and EM noises were combined using random weights α, β, and γ under the constraint α+β+γ=1, and the resulting noisy ECG was generated as

(4) $$x\left(n\right)=s\left(n\right)+K\left[\alpha \eta_{\mathrm{BW}}\left(n\right)+\beta \eta_{\mathrm{MA}}\left(n\right)+\gamma \eta_{\mathrm{EM}}\left(n\right)\right],$$

where *K* scales the noise to the desired SNR [14].

**Figure 3:**
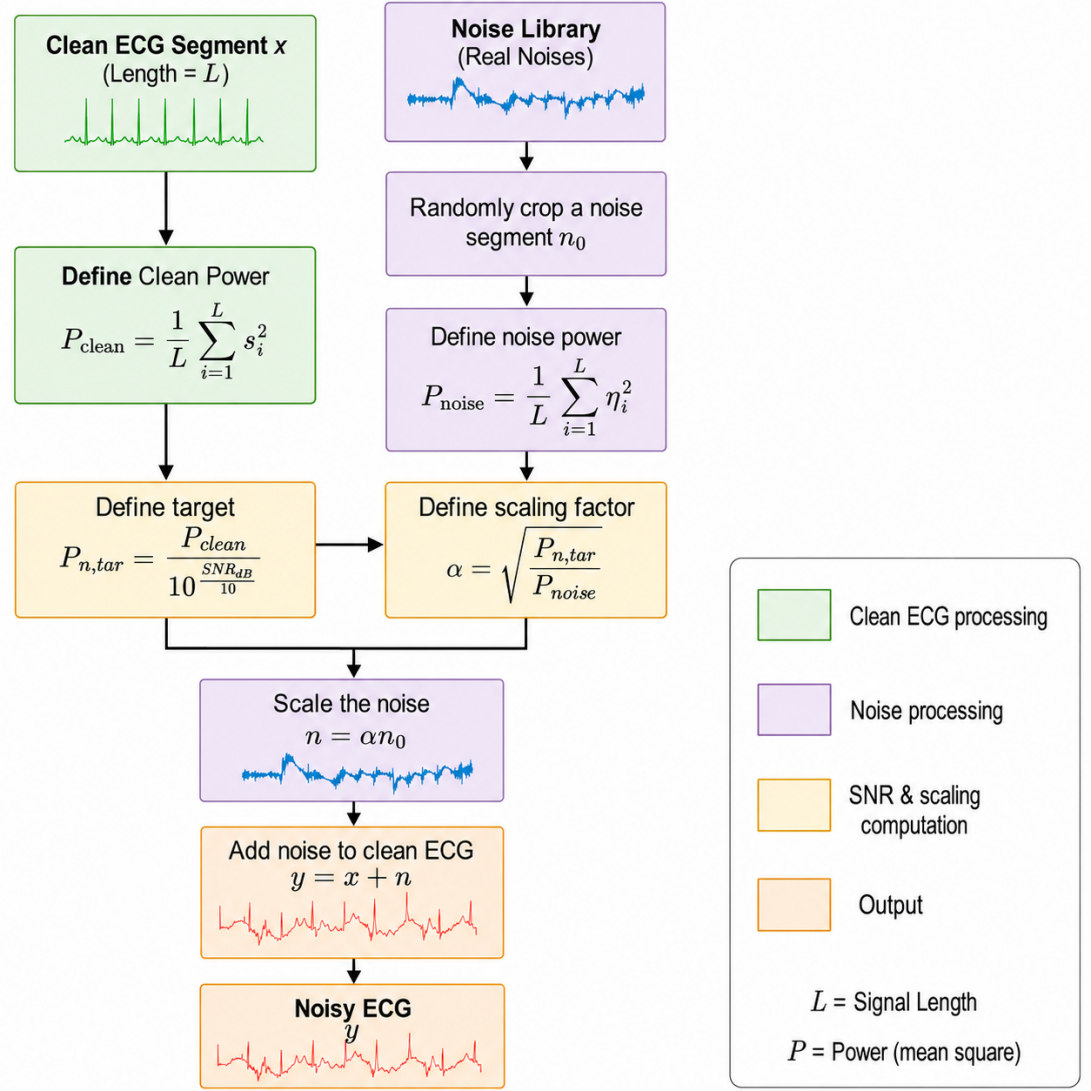
Procedure for real-noise superposition and SNR-controlled noisy ECG generation.

For real-noise construction, a segment of noise with the same length as the clean ECG was randomly selected and scaled to the target SNR before superposition. Figure 3 shows that by using this procedure, noisy ECG samples with varying noise intensity were generated within the specified SNR range, enabling realistic evaluation under wearable recording conditions [38].

### 3.2. Evaluation Metrics

To evaluate the proposed model, DCPA-SNN, on ECG denoising tasks, we consider three metrics: signal-to-noise ratio (SNR), R-peak detection rate, and energy consumption estimation.

#### 3.2.1. Signal-to-Noise Ratio (SNR)

The SNR quantifies the ratio between signal power and reconstruction error. For a reference clean ECG *x* and denoised signal $\hat{x}$:

(5) $$\mathrm{SNR}=10\log_{10}\frac{\sum_{i=1}^{N}x_{i}^{2}}{\sum_{i=1}^{N}{\left(x_{i}-{\hat{x}}_{i}\right)}^{2}}.$$

SNR improvement is computed as:

(6) $${\mathrm{SNR}}_{\mathrm{imp}}={\mathrm{SNR}}_{\mathrm{denoised}}-{\mathrm{SNR}}_{\mathrm{noisy}}.$$

Higher values indicate better noise suppression.

#### 3.2.2. R-Peak Detection Rate

R peaks are critical to heart rate estimation and arrhythmia detection. We detect candidate R peaks on denoised ECG using the Pan–Tompkins algorithm and match them with annotated references from MIT-BIH [26,41]. The detection rate is:

(7) $$\mathrm{DR}=\frac{TP}{TP+FN}\times 100\%,$$

where TP is the number of correctly detected R peaks and FN is the number of missed peaks.

#### 3.2.3. Energy Consumption Estimation

We estimate inference energy based on spike activity [42,43]. Let *r* be the overall spike firing rate:

(8) $$r=\frac{\mathrm{Total}\;\mathrm{Spikes}\;(\mathrm{all}\;\mathrm{LIF}\;\mathrm{layers},\;\mathrm{all}\;\mathrm{timesteps})}{\mathrm{Total}\;\mathrm{Neurons}\;(\mathrm{all}\;\mathrm{LIF}\;\mathrm{layers})}.$$

The effective computational cost for the SNN is:

(9) $${\mathrm{TotalOP}}_{\mathrm{SNN}}=r\times {\mathrm{TotalOP}}_{\mathrm{ANN}}.$$

The theoretical energy consumption for the ANN and SNN is estimated as follows:

(10) $$\begin{array}{cc} E_{\mathrm{ANN}} & ={\mathrm{TotalOP}}_{\mathrm{ANN}}\times E_{\mathrm{MAC}},\;\;\;\;\;\;\;\;\;\;\;\;\;\;\; \end{array}$$

(11) $$\begin{array}{cc} E_{\mathrm{SNN}} & =c\times {\mathrm{TotalOP}}_{\mathrm{ANN}}\times E_{\mathrm{MAC}}+\left(1-c\right)\times r\times {\mathrm{TotalOP}}_{\mathrm{ANN}}\times E_{\mathrm{ADD}}, \end{array}$$

where *c* is the proportion of non-spiking continuous operations, and $E_{\mathrm{MAC}}$ and $E_{\mathrm{ADD}}$ denote the energy of a single MAC and ADD operation, respectively [42].

### 3.3. General Hyperparameters and Computing Platform

**Figure 4:**
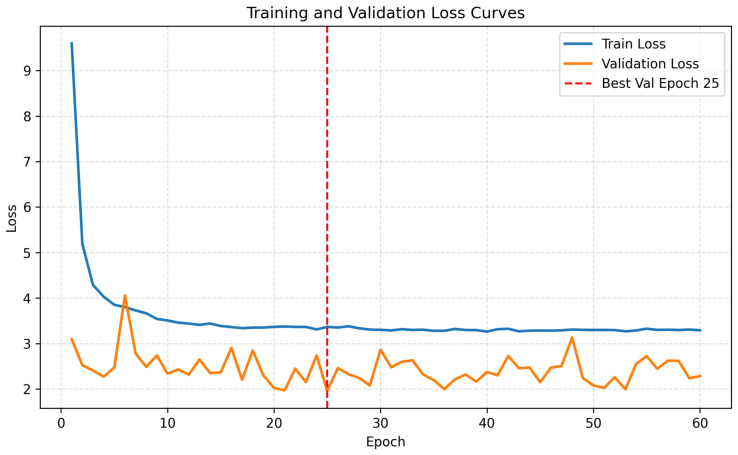
Training and validation loss curves.

The same training hyperparameters were adopted for all deep learning models in this study. Each model was optimized using the Adam optimizer with an initial learning rate of 0.0005 and a batch size of 32. The number of training epochs was set to 60. The training and validation loss curves are shown in Figure 4. A learning-rate scheduler was employed to adaptively adjust the learning rate. Specifically, the learning rate was reduced by a factor of 0.5 when the validation loss did not improve for three consecutive epochs. All experiments, including training, validation, and testing, were conducted on a system equipped with an NVIDIA GeForce RTX 4060 Laptop GPU (8 GB of memory). Python 3.11 was used as the programming environment, with PyTorch 2.5.1 as the primary deep learning framework and SpikingJelly used to implement the SNNs and LIF neurons with surrogate-gradient learning.

## 4. Results and Discussion

### 4.1. Ablation Experiments

The ablation results shown in Table 2 demonstrate that channel attention, residual learning, and the physics-aware loss contribute complementary functions to the proposed model, DCPA-SNN. Removing the channel attention module reduced the average SNR improvement from 6.80 dB to 4.44 dB, corresponding to a decrease of 2.36 dB. The standard deviation also increased from 3.09 dB to 3.81 dB. However, the R-peak detection rate without attention was 95.97%, marginally higher than the 95.72% obtained by the complete model. Given the small absolute difference of 0.25 percentage points and the absence of uncertainty estimates for this metric, this result should not be interpreted as evidence that removing attention improves clinical feature preservation. Rather, it suggests that the principal contribution of channel attention lies in improving global noise suppression and reconstruction stability, whereas R-peak localization is comparatively insensitive to small residual waveform differences.

Residual learning was the most critical architectural component. The negative SNR improvement indicates that direct signal reconstruction without the residual formulation introduced greater reconstruction error than that present in the noisy input.

Removing the physics-aware loss reduced the average SNR improvement from 6.80 dB to 5.63 dB and decreased the R-peak detection rate from 95.72% to 92.13%. The reduction in R-peak detection was proportionally more evident than the decrease in global SNR improvement, indicating that the physics-aware loss contributes particularly to the preservation of clinically important local morphology. Unlike a uniform reconstruction loss, the proposed objective assigns greater importance to QRS, ST, and P-wave regions and incorporates constraints on frequency-domain consistency, QRS slope, R-peak amplitude, differential smoothness, and total variation.

### 4.2. Sensitivity Analysis of Loss-Weighting Coefficients

To investigate the influence of clinically related loss-weighting coefficients, a sensitivity analysis was conducted by varying the R-peak amplitude preservation weight ($\lambda_{r}$) and the QRS slope preservation weight ($\lambda_{qrs}$). Other coefficients were fixed to the values described in Section 2.3.3. Considering the considerable computational cost of retraining the complete SNN model for exhaustive parameter search, only representative configurations covering lower, selected, and higher weighting levels were evaluated.

The results are summarized in Table 3. Moderate variations in $\lambda_{r}$ and $\lambda_{qrs}$ resulted in only limited performance fluctuations, indicating that the proposed model is relatively robust to reasonable changes in loss-weighting coefficients. The optimal configuration ($\lambda_{r}=2.0$ and $\lambda_{qrs}=0.3$) achieved the best overall trade-off between denoising performance and clinical feature preservation. Increasing these coefficients beyond this level did not provide further improvement, suggesting that excessive emphasis on local morphology constraints may introduce unnecessary optimization restrictions.

### 4.3. Denoising Performance Under Single-Noise Conditions

Table 4, Table 5 and Table 6 summarize the denoising performance under muscle artifact, electrode motion artifact, and baseline wander, respectively. Overall, DCPA-SNN achieved significant SNR improvements even under extremely noisy conditions while maintaining high R-peak detection rates. These results indicate that the proposed model effectively suppresses noise while preserving clinically important ECG structures such as the QRS complex. The results are presented separately for three distinct noise conditions.

#### 4.3.1. Denoising Performance Results Under Muscle Artifact (MA) Noise Conditions

**Figure 5:**
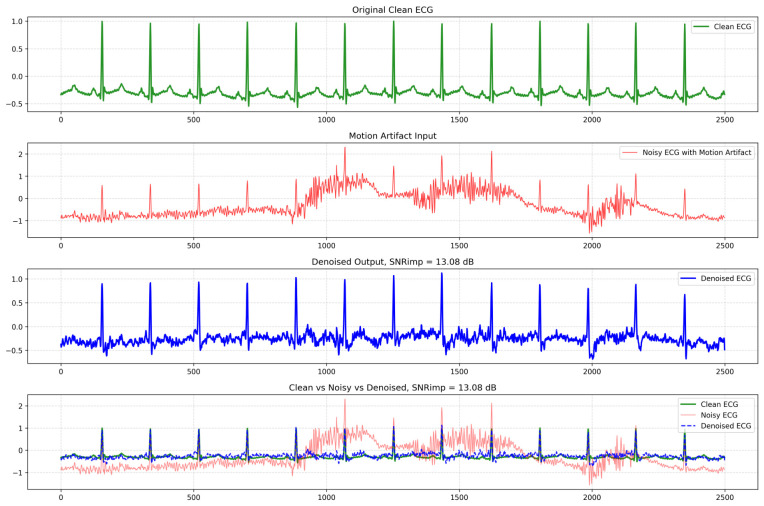
Representative best-case denoising result under muscle artifact noise.

As shown in Figure 5 and Table 4, DCPA-SNN exhibits consistent denoising performance under the condition of muscle artifact noise. Within the entire tested signal-to-noise ratio range, the average denoised SNR is 5.78 dB, the average SNR improvement reaches 6.78 dB, and the average R-peak detection rate is 98.90%. Under low-SNR conditions, the model achieved particularly strong enhancement. When the input SNR ranged from −6 dB to −1 dB, the average SNR improvement reached 8.94 dB. At −6 dB and −4 dB, the SNR improvements were 12.01 dB and 11.50 dB, respectively, with R-peak detection rates of 98.67% and 100.00%. This suggests that DCPA-SNN is effective in handling abrupt and high-frequency interference caused by muscle activity.

Although the overall SNR improvement shows a downward trend in the medium and high signal-to-noise ratio range, the average SNR improvement within this range is 4.20 dB, and the R-peak detection rate reaches 100.00%. This further indicates that the model can still maintain stable denoising performance.

It is worth noting that when the input SNR is −2 dB, the R-peak detection rate drops to 93.89%, which is lower than that at most other signal-to-noise ratio points. This result may be attributed to the high transient amplitude of some muscle artifact segments under this signal-to-noise ratio condition, whose local waveforms may overlap with the QRS complex. When muscle artifacts are temporally close to the R-peak region, noise may alter the amplitude and slope of the QRS complex, leading to distortion and misjudgment of R peaks in the waveform after denoising. Nevertheless, even under this condition, the model still achieves an SNR improvement of 5.52 dB, suggesting that the model maintained effective signal reconstruction under this condition.

According to the experimental results, DCPA-SNN demonstrates effective reconstruction performance in the muscle artifact denoising task. This phenomenon may be attributed to the following mechanism: when transient interference induced by motion is present in the input ECG signals, LIF neurons are capable of capturing dynamic variation features via state evolution along the temporal dimension, which further improves the model’s performance in characterizing non-stationary noise. Compared with conventional continuous-valued convolutional networks, this multi-time step spiking computation mechanism could be better suited for handling muscle artifacts with abrupt features.

#### 4.3.2. Denoising Performance Results Under Electrode Motion (EM) Noise Conditions

Table 5 further illustrates the denoising performance under electrode motion artifact noise. The results show that DCPA-SNN achieved an average SNR improvement of 6.67 dB and maintained an average R-peak detection rate of 96.72%. The improvement was more prominent in the low-SNR range. Specifically, from −6 dB to −1 dB, the average SNR improvement was 8.84 dB, whereas from 0 dB to 4 dB, the average SNR improvement decreased to 4.26 dB. This trend suggests that DCPA-SNN is especially effective when EM noise severely degrades the ECG signal, while the improvement becomes more moderate when the input signal quality is already relatively high.

In terms of clinical feature preservation, the model maintained a high average R-peak detection rate of 96.72% under EM noise. The R-peak detection rate reached 100.00% at 0 dB, 1 dB, 2 dB, 3 dB, and 4 dB. Although the detection rate decreased at −6 dB, −5 dB, −4 dB, −3 dB, −2 dB, and −1 dB, the overall results still demonstrate that the model can preserve most R-peak locations and QRS morphology after denoising. This indicates that DCPA-SNN improves signal quality while maintaining clinically important ECG features.

**Figure 6:**
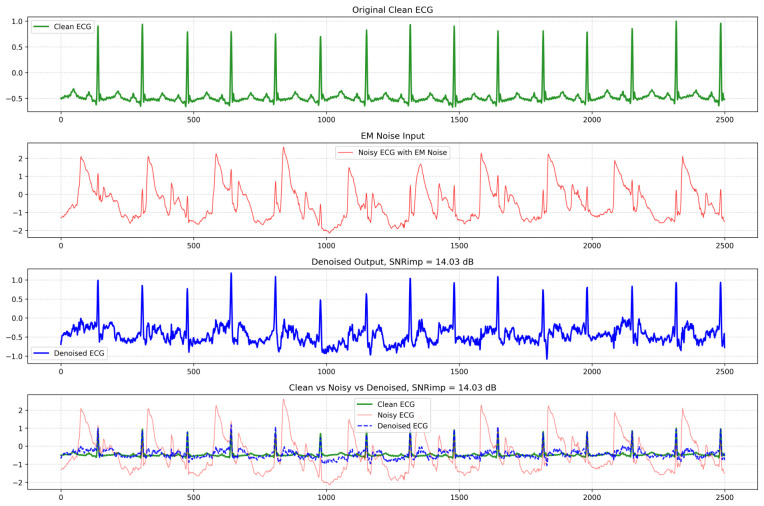
Representative best-case denoising result under electrode motion artifact noise.

As shown in Figure 6, electrode motion artifact often manifests as large-amplitude periodic or abrupt distortions that relate to changes in electrode–skin contact conditions. Due to the SNN’s event-driven temporal modeling mechanism, the LIF neurons accumulate membrane potential over multiple time steps and generate spikes only when the membrane potential exceeds the firing threshold. This mechanism allows the model to respond selectively to meaningful temporal patterns in the ECG signal while suppressing irregular fluctuations. Compared with ordinary continuous-valued convolutional models, the multi-step spiking mechanism provides an additional temporal dimension.

#### 4.3.3. Denoising Performance Results Under Baseline Wander (BW) Noise Conditions

The experimental results show that DCPA-SNN achieved consistent signal quality improvement under all tested BW noise levels. Across all tested SNR levels, the average denoised SNR was 4.04 dB, and the average SNR improvement was 5.04 dB, slightly lower than that obtained under MA (6.78 dB) and EM (6.67 dB) noise. This is reasonable because baseline wander mainly appears as low-frequency drift and may overlap with useful low-frequency ECG components, such as the ST segment and T wave. Therefore, BW removal requires a more conservative reconstruction process to avoid excessive suppression of clinically meaningful morphology.

Despite this challenge, the R-peak detection rate remained above 93% across all tested SNR levels, and the average R-peak detection rate was 96.87% under BW noise. The high R-peak detection rate indicates that DCPA-SNN preserves the main diagnostic structure of ECG signals during BW removal. Although BW mainly affects the low-frequency baseline rather than directly corrupting the sharp QRS complex, large baseline shifts can still influence R-peak localization by changing the local amplitude distribution of the ECG signal. The results show that the proposed model effectively reduces baseline fluctuation while keeping R peaks distinguishable.

**Figure 7:**
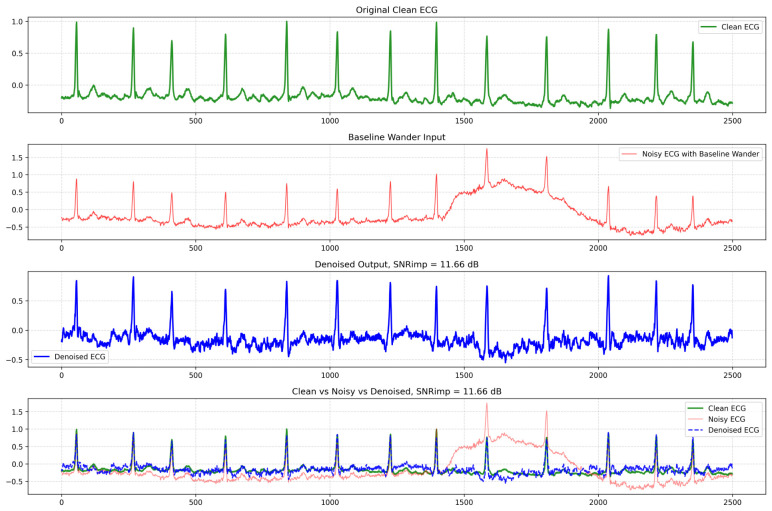
Representative best-case denoising result under baseline wander noise.

As shown in Figure 5, Figure 6 and Figure 7, the denoised waveforms are generally closer to the clean ECG signals than the noisy inputs. The reconstructed signals preserve the major QRS morphology while reducing noise-induced amplitude fluctuations and baseline distortion. These visual results are consistent with the quantitative improvements reported in Table 4, Table 5 and Table 6.

### 4.4. Performance Under Mixed-Noise Conditions

**Figure 8:**
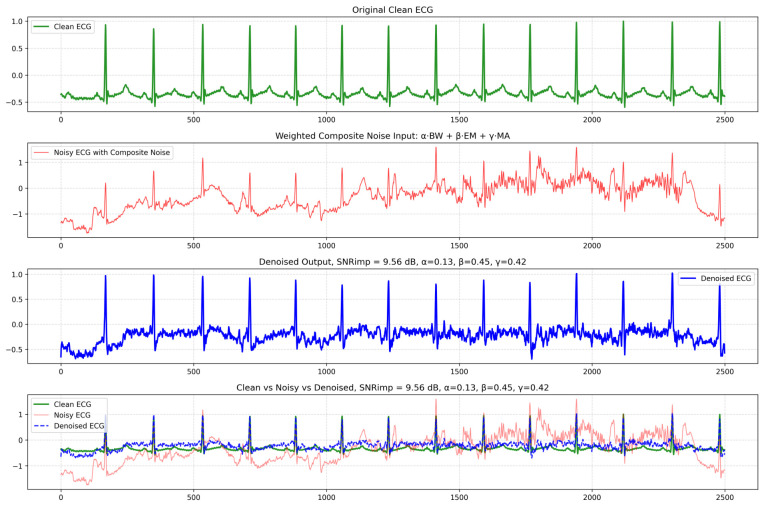
Representative best-case denoising result under mixed-noise conditions.

To further evaluate the robustness of the proposed model, DCPA-SNN, in complex noise environments, mixed-noise experiments were conducted using three standard noise types from the MIT-BIH Noise Stress Test Database, including muscle artifact (MA), electrode motion (EM), and baseline wander (BW). In this experiment, the three noise components were combined using random weighting coefficients to simulate more realistic wearable ECG acquisition conditions, where multiple noise sources may occur simultaneously. The input SNR was varied from −6 dB to 4 dB. The results are shown in Table 7. A representative best-case denoising result under mixed-noise conditions is shown in Figure 8.

The experimental results show that DCPA-SNN achieved stable denoising performance under mixed-noise conditions. Across all tested SNR levels, the average denoised SNR reached 5.80 dB, and the average SNR improvement was 6.80 dB. Compared with the single-noise experiments, the mixed-noise scenario achieved comparable or even slightly higher average SNR improvement. This suggests that DCPA-SNN is not limited to a specific noise pattern but can generalize well to composite noise distributions.

The improvement was more evident under severe noise contamination. In the low-SNR range from −6 dB to −1 dB, the average SNR improvement reached 8.82 dB, whereas in the range from 0 dB to 4 dB, it decreased to 4.38 dB. This trend indicates that DCPA-SNN provides stronger enhancement when the original ECG signal is more severely degraded. As the input signal quality improves, the available margin for further SNR enhancement naturally becomes smaller. This finding again confirms that the proposed model is particularly effective for severely contaminated ECG signals.

In addition to signal reconstruction, Table 7 further compares the R-peak detection rates before and after denoising under mixed-noise conditions. The average R-peak detection rate of the noisy ECG signals was 90.71%, whereas the average detection rate after DCPA-SNN denoising increased to 95.72%, corresponding to an absolute improvement of 5.01 percentage points. This improvement demonstrates that DCPA-SNN not only suppresses mixed noise but also enhances the detectability of clinically important QRS complexes. The improvement was more pronounced under low-SNR conditions. In the range from −6 dB to −1 dB, the average R-peak detection rate increased from 86.07% before denoising to 93.60% after denoising, with an average improvement of 7.53 percentage points. In particular, at −6 dB, the R-peak detection rate increased from 74.82% to 88.55%, and at −4 dB, it increased from 87.90% to 97.24%. These results indicate that DCPA-SNN can substantially recover R-peak information when mixed noise strongly masks the QRS complex. This is especially important for wearable ECG monitoring, where severe motion artifacts and electrode-contact disturbances frequently occur.

In the moderate-SNR range from 0 dB to 4 dB, the average R-peak detection rate increased from 96.50% to 98.34%. Although the improvement in this range was smaller than that in the low-SNR range, the denoised signals maintained consistently high clinical feature detectability. The R-peak detection rate reached 100.00% at 1 dB and 3 dB and remained above 96% at 4 dB. It is worth noting that at −2 dB, the R-peak detection rate after denoising was slightly lower than that of the noisy input, decreasing from 88.78% to 86.85%. This isolated degradation may be related to the complex superposition of baseline wander, electrode motion artifact, and muscle artifact around the QRS region. When transient noise overlaps with R peaks, the denoising process may partially smooth or attenuate local QRS slopes, resulting in missed or shifted R-peak detections. Nevertheless, the model still achieved an SNR improvement of 7.08 dB at this SNR level, indicating that the overall signal reconstruction quality remained strong.

The results in Table 8 show that the model provided a certain degree of waveform protection in the QRS and ST/P regions. The MIT-BIH Arrhythmia Database provides beat-level annotations and R-peak locations, but it does not provide comprehensive manual delineations of P-wave, ST-segment, and T-wave boundaries. Therefore, the present QRS-region and ST/P-region SNR analyses were performed using R-peak-centered clinical time windows rather than manually annotated P/ST/T boundaries. These results provide preliminary evidence of local waveform protection, but future work should validate P-wave, ST-segment, and T-wave preservation using waveform-delineation databases such as the QT Database.

As shown in Table 9, the bootstrap analysis further showed that the SNR improvement was 6.80 ± 3.09 dB with a 95% confidence interval of [6.07, 7.47] dB, while the 95% confidence interval of the R-peak detection improvement was [2.86, 7.42] percentage points. Since this interval did not include zero, the improvement in R-peak detection was statistically supported. We note that the relatively wide confidence intervals are due to record-level bootstrap resampling, which provides a conservative estimate of uncertainty by accounting for inter-record variability.

**Figure 9:**
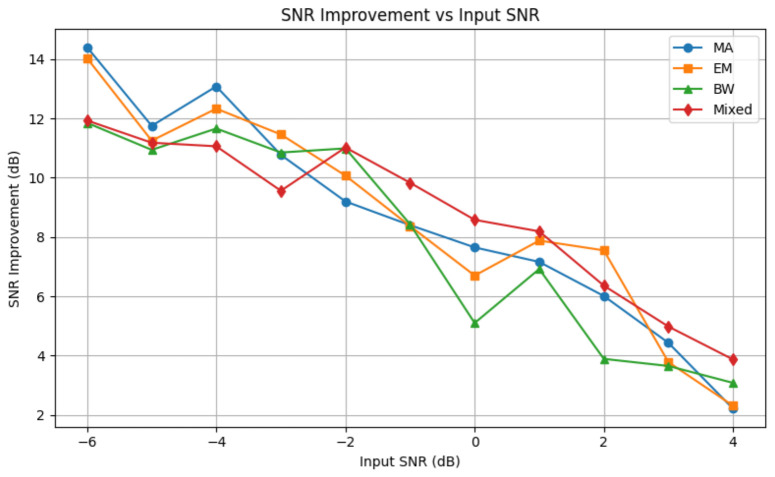
Comparison of average SNR improvement under different noise conditions.

As shown in Figure 9, among the four noise categories, the mixed-noise scenario achieved the highest average SNR improvement of 6.80 dB, followed by muscle artifact (6.78 dB), EM noise (6.67 dB), and baseline wander (5.04 dB). Moreover, the low-SNR range (−6 dB to −1 dB) consistently exhibited larger SNR gains than the high-SNR range (0 dB to 4 dB). This observation indicates that DCPA-SNN is particularly effective for denoising severely contaminated ECG recordings, which are common in wearable monitoring systems.

Beyond R-peak detection metrics, preserving the pathological morphology of P-waves, T-waves, and ST-segments is vital to cardiological diagnosis. The results in Table 8 show that the model provides a certain degree of waveform protection in the QRS and ST/P regions. Future clinical validation should include cardiologist-reviewed diagnostic consistency analysis to confirm that the denoised ECG remains diagnostically reliable.

### 4.5. Zero-Shot External Validation on INCART

To assess cross-database generalization, DCPA-SNN trained on the MIT-BIH-based dataset was directly evaluated on INCART without retraining, fine-tuning, or checkpoint reselection. The external validation results in Table 10 show that the model still achieved positive SNR improvement under severe noise conditions, particularly from −6 dB to 0 dB.

In addition, R-peak detection performance did not consistently improve after denoising on INCART. Although sensitivity remained acceptable in some cases, the number of false-positive detections increased after denoising. As shown in Table 11, the average R-peak detection rate decreased after denoising in the zero-shot INCART validation. The mean detection rate decreased from 98.80% before denoising to 94.79% after denoising. This result suggests that although DCPA-SNN retained partial noise-suppression capability on INCART, its zero-shot preservation of R-peak morphology was limited under cross-database conditions. This discrepancy indicates a clear cross-database morphology shift. INCART contains ECG signals with different acquisition conditions and more heterogeneous waveform morphologies. Even when only annotated normal-beat segments were selected, the QRS polarity, amplitude distribution, local morphology, and baseline characteristics could still differ from the MIT-BIH training distribution. Further multi-database training, domain adaptation, and real wearable ECG validation are required.

Therefore, the proposed model, DCPA-SNN, should be interpreted as a promising wearable-oriented denoising framework under controlled dynamic-noise simulations, rather than a fully validated clinical deployment system.

### 4.6. Theoretical Energy Consumption Evaluation Based on Spike Activity

In addition to denoising performance and waveform preservation, energy efficiency is essential to wearable ECG monitoring systems with limited battery capacity and computational resources. Therefore, the inference energy consumption of DCPA-SNN was evaluated to assess its deployment feasibility.

An isomorphic ANN counterpart was constructed using the same convolutional, deconvolutional, attention, skip-connection, and residual denoising structures. The LIF neurons in DCPA-SNN were replaced with conventional activation functions while maintaining identical network topology. Therefore, the ANN counterpart provides a consistent baseline for energy comparison.

As summarized in Table 12, the input ECG segment length was 1500 sampling points, and the SNN simulation time step was set to T=8. The isomorphic ANN required 60.864 M MACs for one forward inference. Since DCPA-SNN adopts direct coding, the first convolutional layer directly processes continuous ECG inputs and is considered a non-spiking operation. The proportion of non-spiking computation was calculated as c=0.004. Based on the spike activity of all LIF layers across all time steps, the overall spike firing rate was calculated as r=0.68738896, resulting in an effective computational cost of 41.91 M operations.

As summarized in Table 13, the theoretical inference energy was estimated under the 45 nm CMOS setting, where one 32-bit MAC operation consumes 4.6 pJ and one 32-bit ADD operation consumes 0.9 pJ. For the isomorphic ANN, all operations are treated as continuous MAC operations. For DCPA-SNN, the non-spiking computation was calculated using MAC energy, while the spike-driven computation was calculated using ADD energy. Based on the measured spike statistics and operation counts, the theoretical inference energy of DCPA-SNN was estimated at 38.62 µJ, compared with 279.69 µJ for the isomorphic ANN. This corresponds to an energy efficiency improvement of approximately 7.25×.

The energy reduction is mainly attributed to the sparse event-driven computation of DCPA-SNN. Unlike conventional ANNs that perform dense MAC operations regardless of signal activity, DCPA-SNN activates computation only when membrane potentials exceed the firing threshold. This spike-driven mechanism reduces redundant operations by replacing dense MAC computation with sparse ADD operations while maintaining denoising performance.

Although the corrected overall spike firing rate is relatively high because it accumulates spike activity across eight time steps, DCPA-SNN still achieves a 7.25-fold energy reduction compared with its isomorphic ANN counterpart. This conservative spike-activity-based estimation provides a more reliable assessment of SNN energy consumption by considering spike accumulation over the complete inference process.

Overall, under the mixed-noise condition, DCPA-SNN achieves a favorable balance between denoising performance and computational efficiency. Its theoretical inference energy of 38.62 µJ is substantially lower than the 279.69 µJ required by the isomorphic ANN, demonstrating the potential of the proposed event-driven denoising framework for low-power wearable ECG monitoring and neuromorphic edge-device deployment.

### 4.7. Performance Comparison with Representative ECG Denoising Methods

To further evaluate the effectiveness of the proposed method, a comparison with four representative ECG denoising methods for portable wearable devices, including DCAE, VFCDM, R-CED, and the mixed method of DWT, SWT, NLM, and IIR COMB, was conducted. As shown in Table 14, DCPA-SNN achieved an average output SNR of 5.80 dB, an average SNR improvement of 6.80 ± 3.09 dB, and an average R-peak detection rate of 95.72% in the mixed-noise scenario. Compared with DCAE, which achieved an SNR improvement of 5.90 dB and an R-peak precision of 90.00%, DCPA-SNN demonstrated better preservation of clinically important R-peak features. Although VFCDM reported a higher SNR improvement of 10.67 dB, its R-peak detection performance was 92.95%, which was not as accurate as that of DCPA-SNN. R-CED achieved an average SNR improvement of 7.43 dB, and its R-peak detection performance was 91.86%, which was also not as accurate as the proposed method.

In addition, the study by Kozłowski et al. investigated the effects of several traditional denoising strategies, including DWT, SWT, EMD-DWT, VMD-DWT, NLM, EMD-NLM, and IIR COMB, on R-peak detection under different noise conditions. Their results showed that appropriate denoising strategies can improve autonomous R-peak detection. Since that study reported R-peak sensitivity and accuracy rather than the same average R-peak detection rate used in this work, its result is included as a reference comparison. Based on the best-performing strategies across the reported noise types, the average R-peak detection accuracy was approximately 96.97%. This result further confirms that R-peak preservation is an important criterion for evaluating ECG denoising methods.

Overall, these comparisons indicate that DCPA-SNN provides a favorable balance between noise suppression and R-peak preservation. Compared with traditional denoising methods, DCPA-SNN offers an end-to-end event-driven denoising framework and further considers electrode motion artifact and mixed-noise conditions, which are more representative of dynamic wearable ECG monitoring. Moreover, the proposed method also provides low-power deployment potential due to the sparse spike firing mechanism of SNNs.

Since different studies used different datasets, noise settings, and evaluation protocols, the comparison should be interpreted as a reference rather than a strictly controlled benchmark. Cross-paper comparisons cannot be considered strictly fair unless the same dataset, protocol and metrics are used.

### 4.8. Limitations

While the proposed framework, DCPA-SNN, achieves stable denoising and low theoretical energy consumption, its evaluation carries notable limitations. Primarily, the model was tested chiefly on normal heartbeats to establish a baseline for physics-guided learning. Its robustness on complex pathological waveforms, such as premature ventricular contractions (PVCs) and bundle branch blocks, remains unverified—particularly in low-SNR environments where abnormal QRS complexes and transient artifacts overlap. Additionally, using artificially superimposed noise fails to replicate real-world wearable dynamics, such as time-varying impedance drift and sweat-induced contact degradation. Therefore, prospective validation on real wearable devices is strictly required prior to clinical or commercial deployment. In addition, because MIT-BIH does not provide comprehensive P-wave, ST-segment, and T-wave boundary annotations, future work should validate morphology preservation using dedicated waveform-delineation databases.

Future work will utilize diverse, annotated arrhythmia databases to confirm the model’s diagnostic consistency across various abnormal-beat types. We also plan to implement a real-world validation protocol to collect wearable ECG signals under diverse daily activities, including resting, walking, arm movements, and postural changes. The model’s practical utility will be benchmarked via SNR improvement, R-peak accuracy, waveform correlation, morphology preservation indices, and motion-state failure analysis. Lastly, lightweight post-processing filters and morphology constraints will be investigated to suppress residual spike-like artifacts while preserving critical diagnostic ECG structures.

## 5. Conclusions

In this work, Direct-Coding-Physics-Aware Spiking Neural Network, or DCPA-SNN, was proposed for wearable ECG denoising under dynamic-noise conditions encountered in wearable monitoring. The proposed method treats ECG denoising as an event-driven signal reconstruction task and integrates a multi-step SNN encoder–decoder architecture, bottleneck channel attention, residual noise estimation, and a physics-aware multi-domain loss function. By combining direct coding with the temporal integration and threshold-triggered firing characteristics of LIF neurons, the model provides a potential pathway toward low-power ECG denoising. Experimental results on the MIT-BIH Arrhythmia Database and the MIT-BIH Noise Stress Test Database demonstrate that DCPA-SNN performs favorably in terms of both denoising performance and power consumption. Specifically, this study first investigated the model’s denoising performance in terms of SNR and R-peak detection, followed by a theoretical evaluation of its energy consumption. In addition, experiments under single-noise conditions were conducted to evaluate its capability of suppressing the MA, EM, and BW noise types separately. Finally, comparisons with existing methods suggest that the proposed DCPA-SNN achieves a favorable trade-off between noise suppression and power consumption. These findings suggest that DCPA-SNN has promising potential for low-power wearable ECG monitoring and neuromorphic edge-device deployment, while the actual system-level advantage must be verified through embedded hardware or neuromorphic-platform implementation. This work establishes the feasibility of the proposed SNN framework for foundational ECG denoising, paving the way for larger-scale validation on complex pathological morphologies prior to clinical deployment.

## Figures and Tables

**Table 1:** Types of ECG noise and their characteristics.

Noise Type	Source	Effect on ECG	Example
Baseline Wander (BW)	Respiration, electrode movement, etc.	Low-frequency drift of baseline	Slow baseline shifts
Muscle Artifact (MA)	Patient movement, electrode displacement, etc.	Distorted waveform, amplitude fluctuation, etc.	Sudden spikes during motion
Electrode Motion (EM)	Poor skin contact, sweat, etc.	Sharp transient noise	Abrupt voltage jumps

**Table 2:** Ablation study of the proposed model components.

Configuration	SNR Improvement (dB)	R-Peak Detection (%)
Proposed	6.80 ± 3.09	95.72
Without attention	4.44 ± 3.81	95.97
Without residual learning	−1.55 ± 1.11	56.97
Without physics-aware loss	5.63 ± 3.76	92.13

**Table 3:** Sensitivity analysis of clinically related loss-weighting coefficients.

Configuration	$\lambda_{r}$	$\lambda_{\mathrm{qrs}}$	SNR Improvement (dB)	R-Peak Detection (%)
A	1.0	0.3	5.34 ± 3.81	94.62
B	2.0	0.3	6.80 ± 3.09	95.72
C	3.0	0.3	5.90 ± 3.66	95.48
D	2.0	0.1	6.78 ± 3.46	95.11
E	2.0	0.5	6.42 ± 3.15	95.48

**Table 4:** Denoising performance under MA noise.

Input SNR (dB)	Denoised SNR (dB)	SNR Improvement (dB)	95% CI (dB)	R-Peak Detection (%)
−6	6.01±1.75	12.01±1.75	[10.60, 13.44]	98.67
−5	3.96±3.04	8.96±3.04	[6.50, 11.25]	97.33
−4	7.50±0.96	11.50±0.96	[10.86, 12.33]	100.00
−3	6.73±1.28	9.73±1.28	[8.64, 10.58]	100.00
−2	3.52±3.46	5.52±3.46	[2.88, 8.16]	93.89
−1	4.89±2.17	5.89±2.17	[4.06, 7.42]	98.00
0	6.40±0.73	6.40±0.73	[5.93, 7.07]	100.00
1	5.79±2.00	4.79±2.00	[3.28, 6.31]	100.00
2	6.52±1.59	4.52±1.59	[3.26, 5.62]	100.00
3	6.55±0.62	3.55±0.62	[3.08, 4.05]	100.00
4	5.75±0.93	1.75±0.93	[1.11, 2.50]	100.00
Average	5.78	6.78	-	98.90

**Table 5:** Denoising performance under EM noise.

Input SNR (dB)	Denoised SNR (dB)	SNR Improvement (dB)	95% CI (dB)	R-Peak Detection (%)
−6	6.42±1.16	12.42±1.16	[11.52, 13.33]	85.71
−5	4.40±1.88	9.40±1.88	[7.80, 10.67]	99.05
−4	7.01±1.44	11.01±1.44	[9.84, 12.10]	98.67
−3	5.75±3.23	8.75±3.23	[5.95, 10.91]	96.00
−2	3.86±4.16	5.86±4.16	[2.56, 9.17]	93.39
−1	4.60±2.92	5.60±2.92	[3.32, 7.88]	91.11
0	6.21±0.56	6.21±0.56	[5.77, 6.64]	100.00
1	5.86±2.04	4.86±2.04	[3.37, 6.38]	100.00
2	7.29±2.05	5.29±2.05	[3.77, 6.81]	100.00
3	6.40±0.36	3.40±0.36	[3.11, 3.65]	100.00
4	4.52±2.08	0.52±2.08	[−0.49, 1.53]	100.00
Average	5.67	6.67	-	96.72

**Table 6:** Denoising performance under BW noise.

Input SNR (dB)	Denoised SNR (dB)	SNR Improvement (dB)	95% CI (dB)	R-Peak Detection (%)
−6	1.05±3.70	7.05±3.70	[4.14, 9.87]	93.65
−5	3.96±1.83	8.96±1.83	[7.49, 10.36]	94.04
−4	5.59±1.60	9.59±1.60	[8.43, 10.78]	100.00
−3	4.91±2.62	7.91±2.62	[5.95, 9.87]	93.75
−2	5.15±3.68	7.15±3.68	[3.91, 9.61]	100.00
−1	4.37±3.01	5.37±3.01	[3.13, 7.60]	98.95
0	2.57±1.90	2.57±1.90	[1.06, 4.12]	95.81
1	3.65±3.29	2.65±3.29	[0.09, 5.12]	96.19
2	3.81±2.28	1.81±2.28	[0.02, 3.60]	94.67
3	4.11±3.01	1.11±3.01	[−1.52, 3.25]	98.46
4	5.22±1.21	1.22±1.21	[0.35, 2.24]	100.00
Average	4.04	5.04	-	96.87

**Table 7:** Denoising results under mixed-noise conditions.

Input SNR (dB)	Denoised SNR (dB)	SNR_imp (dB)	Noisy RDet (%)	Denoised RDet (%)
−6	5.04 ± 0.83	11.04 ± 0.83	74.82	88.55
−5	3.46 ± 1.88	8.46 ± 1.88	85.58	93.87
−4	5.89 ± 0.61	9.89 ± 0.61	87.90	97.24
−3	5.90 ± 1.16	8.90 ± 1.16	86.02	96.44
−2	5.08 ± 2.62	7.08 ± 2.62	88.78	86.85
−1	6.52 ± 2.27	7.52 ± 2.27	93.33	98.67
0	5.42 ± 1.03	5.42 ± 1.03	93.21	95.71
1	6.67 ± 1.61	5.67 ± 1.61	95.59	100.00
2	7.73 ± 1.42	5.73 ± 1.42	97.71	100.00
3	5.34 ± 1.31	2.34 ± 1.31	100.00	100.00
4	6.72 ± 1.20	2.72 ± 1.20	96.00	96.00
Average	5.80 (avg)	6.80 (avg)	90.71	95.72

**Table 8:** QRS and ST/P region SNR improvement evaluation.

QRS-Region SNR_imp (dB)	ST/P-Region SNR_imp (dB)	Total SNR_imp (dB)
5.68 ± 3.12	7.91 ± 3.57	6.80 ± 3.09

**Table 9:** Statistical analysis of SNR improvement and R-peak detection under mixed-noise conditions.

Metric	Mean ± SD	95% CI
SNR improvement (dB)	6.80 ± 3.09	[6.07, 7.47]
Noisy R-peak detection (%)	90.71 ± 1.54	[87.56, 93.59]
Denoised R-peak detection (%)	95.72 ± 1.26	[93.03, 97.94]
R-peak detection improvement (percentage points)	5.01 ± 1.19	[2.86, 7.42]

**Table 10:** SNR improvement under different noise conditions.

Noise Type	−6 dB	−5 dB	−4 dB	−3 dB	−2 dB	0 dB	Average
BW	7.82±0.71	7.05±0.77	6.28±0.84	5.46±0.91	4.65±0.98	2.97±1.10	5.71
EM	7.58±0.66	6.85±0.74	6.15±0.84	5.36±0.89	4.57±0.94	2.89±1.04	5.57
MA	7.41±0.74	6.65±0.69	5.88±0.84	5.17±0.88	4.34±0.96	2.75±1.11	5.37
Mixed	7.60±0.64	6.89±0.80	6.09±0.80	5.28±0.86	4.51±0.97	2.87±1.14	5.54

**Table 11:** Average R-peak detection rate before and after denoising in zero-shot INCART validation.

Noise Type	Noisy ECG (%)	Denoised ECG (%)	Change (% Points)
BW	99.86	95.90	−3.96
EM	98.06	95.37	−2.69
MA	98.42	93.19	−5.23
Mixed	98.87	94.71	−4.17
Average	98.80	94.79	−4.01

**Table 12:** Statistical settings used for DCPA-SNN energy estimation.

Item	Value or Calculation
Time steps *T*	8
Isomorphic ANN	60.864 M MACs
Proportion of non-pulse layers	0.004
Overall pulse rate	0.68738896
SNN FLOPs	41.91 M ADDs

**Table 13:** Theoretical energy comparison between DCPA-SNN and its isomorphic ANN counterpart.

Model	Equivalent Operations	Energy per Operation	Theoretical Energy	Relative Efficiency
Isomorphic ANN	60.864 M MACs	4.6 pJ/MAC	279.69 µJ	1×
DCPA-SNN	41.91 M ADDs	0.9 pJ/ADD	38.62 µJ	7.25×

The estimation uses the 45 nm CMOS setting, where one 32-bit MAC consumes 4.6 pJ and one 32-bit ADD consumes 0.9 pJ.

**Table 14:** Comparison of average SNR, R-peak detection, and energy consumption evaluation among the reviewed methods.

Paper/Source	Method	Avg. SNR Improvement (dB)	Avg. R-Peak Detection (%)	Energy Evaluation
Occhipinti et al. [44]	DCAE	median 5.90	median 90.00	—
Hossain et al. [45]	VFCDM	10.67 ± 5.20	92.95	—
Reljin et al. [46]	R-CED	7.43 ± 0.81	91.86	—
Kozlowski et al. [47]	DWT/SWT/EMD-DWT/VMD-DWT/NLM/IIR COMB	—	96.97	—
Proposed model	DCPA-SNN	6.80 ± 3.09	95.72	38.62 µJ

For DCPA-SNN, all metrics are reported under the mixed-noise scenario (input SNR from −6 dB to 4 dB) and are consistent with Table 7 and Table 9. Denoised SNR and SNR improvement are averaged across the eleven input-SNR levels; R-peak detection is the corresponding average denoised detection rate (95.72%). Other methods are cited from the original papers and are included for reference only.

## Data Availability

The original contributions presented in this study are included in the article. Further inquiries can be directed to the corresponding author.
